# Predictors of Significant Patient Movement During Frameless Radiosurgery with the Gamma Knife® Icon™ Cone-Beam CT

**DOI:** 10.7759/cureus.21380

**Published:** 2022-01-18

**Authors:** William N Duggar, Bart Morris, Rui He, Claus Chunli Yang

**Affiliations:** 1 Radiation Oncology, University of Mississippi Medical Center, Jackson, USA

**Keywords:** motion, hdmm, icon, frameless, gamma knife

## Abstract

Objective

The objective of the study is to discern any factors that may be predictive of patient-specific uncertainty related to residual error after cone-beam CT (CBCT) correction and motion measured by the high-definition motion management (HDMM) system.

Methods

HDMM treatment logs were parsed via a Python 3 script and then analyzed for 30 patients. Additionally, CBCT registration and correction data was also collected and analyzed for the same 30 patients. Correlation analysis was then performed against various patient- and treatment-related factors to discern any potentially predictive factors.

Results

BMI was the only statistically significant predictor identified in this study with an r value of 0.393, p=0.032. Despite being identified as a predictor in other studies, treatment time, when treated as a continuous variable, did not show up as significant in this work.

Conclusion

BMI may be predictive of patients who might require extra tactics to mitigate motion during frameless Gamma Knife® treatment.

## Introduction

The Gamma Knife® Icon™

Elekta introduced the Gamma Knife® Icon™ in 2016 after building on the foundation of the Perfexion™ as a treatment delivery system. The Icon™ includes both a cone-beam CT (CBCT) arm for pre-treatment image guidance and a high-definition motion management (HDMM) system for motion management during treatment delivery. Additionally, the Icon™ makes use of a mask adaptor rather than the traditional G-frame adaptor so that patients may be immobilized by a thermoplastic mask rather than only the stereotactic G-frame [1,2]. Due to these enhancements, the Icon™ allows the Gamma Knife® to compete with other radiosurgery modalities in offering frameless, image-guided, and/or fractionated stereotactic radiotherapy. Several groups have shared their experiences utilizing the Gamma Knife® Icon™ for frameless radiosurgery [3,4].

Frameless Gamma Knife® Icon™ motion management

Prior to treatment, the patient is positioned in the mask adaptor with their own thermoplastic mask, and a temporary nose fiducial is placed. A CBCT is acquired, which is transferred to GammaPlan® and co-registered with a reference CBCT taken during mask creation. The treatment plan is re-calculated and corrected based on the current CBCT, and residual dose differences from the original treatment plan are shown for review by the treatment team. Upon approval, the workflow returns to the treatment console, and the delivery is commenced. The HDMM system will set a baseline based on the average position of the nose fiducial marker relative to reference markers housed on the mask adaptor during the CBCT acquisition. The fiducial marker is then tracked to stay within a customer-defined threshold distance (i.e., 1.5 mm) from the baseline position. Treatment is gated based on the marker position relative to the baseline and only resumes if the total displacement is below the threshold. If the threshold is exceeded and the original position cannot be attained, the CBCT imaging step can be repeated and a new baseline set. This process continues until treatment delivery is completed. Motion below the threshold would also ideally be limited, but should the threshold be set too tight, interruptions to treatment become more likely with each repeat CBCT process adding at least five minutes to the total treatment time. Motion and interruptions should be limited for optimal treatment. To contribute to this effort, identification of patient or treatment-related factors leading to more patient motion or interruptions would be useful in optimizing frameless Gamma Knife® treatment. Others have engaged in similar work, but this work still offers the opportunity to both contribute to the body of knowledge on this topic, validate their results, and also illuminate areas of interest not previously investigated [5,6]. 

## Materials and methods

Patient HDMM and CBCT data

HDMM log files for the treatments of 30 patients were parsed and interpreted by a Python script received from Elekta. These log files record the marker position each time the radial position changes from baseline by more than 0.2 mm. The x, y, and z marker coordinates can also be extracted and compared to the post-CBCT reference position, but it should be noted that x, y, and z are instantaneously recorded, whereas the markers radial position (vector sum of x, y, and z) is averaged over some time (i.e., 500 ms) for its record in the log files. Area under the curve (AUC) analysis was used to calculate the mean position over the treatment relative to the last reference marker position after the most recent CBCT correction. When the marker exceeds the threshold, treatment is paused. Therefore, only marker data during actual treatment delivery (beam-on) was included in the analysis as relevant to treatment accuracy since treatment only resumes after correction or marker movement back within tolerance. Additional technical specifics of the HDMM system can be found in the literature [7].

CBCT imaging and registration that was necessarily repeated during patient treatments was recorded with translational and rotationally applied corrections from baseline reference CBCT. After CBCT correction, the residual setup error post CBCT was also recorded by the system in the aforementioned log files. This HDMM marker data was retrospectively collected and analyzed. The residual radial error after CBCT and mean radial HDMM displacement during treatment was added in quadrature to calculate patient-specific random uncertainty per International Committee for Radiological Units (ICRU) recommendations [8]. This data was analyzed versus various patient-related factors with the statistical analysis to determine potential predictive variables.

Some of the listed variables require further detail in how values were assigned. The disease site was designated as central if it was more than 1 cm from the inner edge of the skull. Body mass index (BMI) was calculated based on the conventional formula. Patient head size was measured at the largest axial dimensions in the MRI scan. The elliptical area was calculated by an assumption of an ellipse of the measured length and width dimensions. Karnofsky performance scale (KPS) was recorded from the clinically assigned value at the time of radiation consult which was typically one to two weeks prior to treatment. Target shape was described by the sphericity ratio of the diameter of an equivalent sphere in volume by the maximum measured diameter of the target in any direction. The other variables are defined simply in Table 1 to a reasonable extent. 

**Table 1:** Variables of interest included in this work GTV - gross target volume

Variable	Type	Values	Units	Category
Patient specific random uncertainty	Continuous ratio	0.45 - 2.39	Millimeters	Dependent
Disease site	Dichotomous nominal	Central, peripheral	N/A	Independent
Patient age	Continuous ratio	36 - 85	Years	Independent
Patient weight	Continuous ratio	112-298	lbs	Independent
Patient height	Continuous ratio	60-76	In	Independent
Patient BMI	Continuous ratio	18.36 - 41.97	N/A	Independent
Patient head size (width and length)	Continuous ratio	13.98 - 21.57	cm	Independent
Patient head elliptical area	Continuous ratio	209.21 - 278.72	cm^2^	Independent
Karnofsky performance scale (KPS)	Continuous ratio	60 - 90	N/A	Independent
Target size	Continuous ratio	0.003 - 26.78	cm^3^	Independent
Fractionation scheme	Continuous ratio	1 to 5	Fractions	Independent
Target shape (equivalent sphere/max diameter)	Continuous ratio	0.366 - 0.863	N/A	Independent
Number of targets	Continuous ratio	1 - 4	N/A	Independent
Treatment time per fraction	Continuous ratio	6.4 - 107.8	Minutes	Independent
Largest GTV	Continuous ratio	0.19 - 26.78	cm^3^	Independent
Total GTV	Continuous ratio	0.19 - 32.30	cm^3^	Independent

## Results

HDMM system

Thirty different patients underwent HDMM monitoring for treatment times between 6.4 and 107.8 min. Sixteen of the patients received treatment in a single fraction while 14 underwent fractionated regimens of three to five treatments for total dose delivery. The average AUC during treatment for all patients was -0.0007 ± 0.28, -0.06 ± 0.24, 0.24 ± 0.27, and 0.72 ± 0.24 mm for x, y, z, and radial, respectively. The 95% CI's for x, y, z, and 3D total are [-0.07, 0.07], [-0.11, 0], [0.17, 0.30], and [0.63, 0.80], respectively. The reported residual HDMM marker position immediately post CBCT correction was averaged to be -0.02 ± 0.17, -0.02 ± 0.21, 0.11 ± 0.37, and 0.45 ± 0.32 mm for x, y, z, and radial, respectively, for all 30 patients. The 95% CI's for x, y, z, and 3D total are [-0.06, 0.02], [-0.08, 0.03], [0, 0.23], and [0.34, 0.57], respectively. These results are in Table 2.

**Table 2:** Correlation analysis with patient-specific uncertainty (HDMM motion and CBCT correction) HDMM - high-definition motion management; CBCT - cone-beam CT; GTV - gross target volume

Variable	Coefficient	p-value	Strength	Significant	95% CI	n
Patient age	0.269	0.151	Weak	No	-0.101, 0.574	30
Patient weight	0.355	0.054	Moderate	No	-0.006, 0.634	30
Patient height	-0.089	0.64	None	No	-0.435, 0.280	30
Patient BMI	0.393	0.032	Moderate	Yes	0.038, 0.660	30
Patient head diameter anterior-posterior (AP)	0.034	0.859	None	No	-0.330, 0.390	30
Patient head diameter left-right (LR)	0.139	0.463	Weak	No	-0.233, 0.475	30
Patient head elliptical area	0.101	0.596	Weak	No	-0.269, 0.445	30
Karnofsky performance scale (KPS)	-0.21	0.264	Weak	No	-0.530, 0.163	30
Individual target size	-0.086	0.536	None	No	-0.346, 0.186	54
# of fractions	-0.355	0.054	Moderate	No	-0.634, 0.006	30
# of shots	0.078	0.682	None	No	-0.290, 0.426	30
Target shape (equivalent sphere/max diameter)	-0.162	0.241	Weak	No	-0.412, 0.111	54
# of targets	0	1	None	No	-0.360, 0.360	30
Treatment time per fraction	0.21	0.265	Weak	No	-0.163, 0.530	30
Largest GTV	-0.154	0.418	Weak	No	-0.487, 0.218	30
Total GTV	-0.086	0.536	None	No	-0.433, 0.283	30

Wright et al. investigated the correlation of the HDMM to the motion of intracranial targets. Generally, intracranial targets were displaced about half that of measurement of the nose fiducial by the HDMM; however, situations did exist where measurement of the nose fiducial did grossly underestimate intracranial motion such as targets located superiorly and patient head rotation around the y-axis [7]. In this work, the HDMM readout was used for an assessment of patient motion in general within the immobilization system and during treatment. This assessment was then seen as a conservative measurement of each patient's target motion since the relationship to the nose marker is dependent on both location and type of movement, which is not as easily discerned as the target location is.

Predictors of motion uncertainty

Since target location was the only nominal variable of investigation, the targets were grouped by either central or peripheral (within 1 cm of the skull), and an independent samples t-test was performed. The statistics results, including an independent samples t-test between groups of 54 targets (41 peripheral, 13 central) from 30 patients, yield a non-significant statistical difference with central M=1.29 (0.57) and peripheral M=1.12 (0.45), t(16.955)=1.115, p=0.305. The 95% CI of the difference of the means was 0.17103 (-0.19615, 0.5382). So, there is no apparent relationship between target location and patient-specific random uncertainty. 

In the bivariate Pearson correlation (see Table 2) analysis for patient-specific random uncertainty, only one factor was found to have a statistically significant correlation with the overall random uncertainty for a given patient. BMI (body mass index) had a moderate positive correlation with patient-specific random uncertainty with an r value of 0.393, p=0.032. This indicates that as BMI increases, the random uncertainty for patients was typically higher for this population. 

## Discussion

There appears to be a possible increase in uncertainty with the increase in patient BMI. This correlation was only moderate, so likely many other factors are at play, but perhaps one could take this relationship into account when making clinical decisions regarding patients with increased BMI. For instance, more intentional coaching during the mask making process to prevent movement so that perhaps the mask will immobilize better. In addition, methods to assist the patient in resting during treatment to prevent movement might also be considered. Why BMI showed up as a predictor for higher patient-specific uncertainty is perhaps an opportunity for further study. One possibility might be the less rigid and more fleshy regions of the face, such as the chin where the Icon™ mask is designed to be reinforced for stability, but when pressing against more soft tissue rather than bone, the mask may not be as effective. Also, the additional mass may push the limitations of the mask's ability to immobilize the head just by simple mechanical forces. Whatever the reason, this was an interesting finding and probably warrants further study.

When comparing this data to the conclusions of others, it seems to add value to their data. Other work identified patient sex, Eastern Cooperative Oncology Group (ECOG) score (which we did not historically record for our patients), pre-treatment rotation, use of anxiolytics, tumor type, and treatment time as important factors to consider [5,6]. This work did not consider any of the aforementioned factors due to limitations of data except for treatment time. Though this work did not find treatment time to be a significant predictor, it was evaluated as a continuous variable in our study, whereas in the other work, it was treated as nominal based on whether less than or greater than 19 min, which could explain the different conclusions. Interestingly, the same study also considered BMI, but only on a nominal basis (overweight or not), whereas again, our work treated BMI as a continuous variable [5]. Had the previous work picked a different threshold or chosen a continuous comparison, the results may have been different. In any case, all of the factors offer insight into how clinicians might improve not only the treatment quality but also the patient experience during frameless Gamma Knife® treatment.

## Conclusions

BMI was identified as a potential predictor of patient-specific uncertainty, which perhaps could be considered during steps like the mask making process or treatment preparation. This conclusion adds to those of previous work identifying total treatment time, use of anxiolytics, age, and even sex as concerns during frameless Gamma Knife® treatment. Perhaps, a pre-simulation screening may lead to strategy implementation to improve immobilization on a per patient basis with frameless Gamma Knife® radiosurgery/therapy. At a minimum, special attention can be given to patients during simulation when these factors are present.
